# Dietary Germinated Paddy Rice and Stocking Density Affect Egg Performance, Serum Biochemical Properties, and Proteomic and Transcriptomic Response of Laying Hens Exposed to Chronic Heat Stress

**DOI:** 10.3390/proteomes9040048

**Published:** 2021-12-13

**Authors:** Tossaporn Incharoen, Sittiruk Roytrakul, Wirot Likittrakulwong

**Affiliations:** 1 Department of Agricultural Science, Faculty of Agriculture Natural Resource and Environment, Naresuan University, Phitsanulok 65000, Thailand; tossaporni@nu.ac.th; 2 National Center for Engineering and Biotechnology (BIOTEC), National Science and Technology Development Agency, Pathumthani 12100, Thailand; sittiruk@biotec.or.th; 3 Animal Science Program, Faculty of Food and Agricultural Technology, Pibulsongkram Rajabhat University, Phitsanulok 65000, Thailand

**Keywords:** germinated paddy rice, stocking density, protein abundance profiles, gene expression, LC-MS/MS

## Abstract

Germinated paddy rice (GPR) could be a good alternative feed source for poultry with stocking density and heat stress problems. A total of 72 Hy-line Brown laying hens raised under low (LSD, 0.12 m^2^/bird) and high stocking densities (HSD, 0.06 m^2^/bird) were investigated. Three dietary GPR levels (0, 74 and 148 g/kg) were used. It was found that average daily feed intake, hen-day egg production, and egg mass significantly decreased in the HSD group. The levels of serum glucose (GLU), phosphorous (P), corticosterone (CORT), total Ig, lysozyme (LZY), and superoxide dismutase activities (SOD) in the HSD group were higher than those in the LSD group. Dietary GPR significantly affected GLU, P, alternative complement haemolytic 50 (ACH50), total Ig, and LZY. Moreover, CORT level significantly decreased in 74 and 148 g/kg dietary GPR groups, whereas SOD significantly increased only in the 148 g/kg dietary GPR group. Serum samples were analyzed using liquid chromatography-tandem mass spectrometry, and 8607 proteins were identified. Proteome analysis revealed 19 proteins which were enriched in different stocking densities and dietary GPR levels. Quantitative real-time reverse transcription-PCR technique was successfully used to verify the differentiated abundant protein profile changes. The proteins identified in this study could serve as appropriate biomarkers.

## 1. Introduction

Heat stress (HS) is the most critical physical stressful factor for the poultry industry. HS is caused by high ambient temperature, nutritional stress (nutritional imbalance), and high stocking density (HSD) [1]. HSD plays a major role in stimulating HS in poultry. Abudabos et al. [2] showed that the body temperature of chickens is raised at HSD and was higher than that in low stocking density (LSD). Studies on the physiological responses of poultry to stocking density suggested that hens reared at HSD had lower egg performance, biochemical parameters, and immune activities [3,4,5,6]. Nutritional factors play an important role in alleviating the stress from HSD in laying hens.

A high moisture content of rice grains leads to their germination. Germinated paddy rice (GPR) has enhanced nutritional qualities. Hydrolytic enzymes and other biological components are activated during germination, while starch, polysaccharides, and amino acids are decomposed. The decomposition of polymers in germinated cereal leads to the generation of functional materials [7]. GPR has many nutritional components, including carbohydrates, protein, oil, dietary fiber, vitamin, γ-oryzanol, and γ-amino butyric acid (GABA) [7]. GABA acts as a primary inhibitory neurotransmitter in the central nervous system of animals [8]. Several reports have shown that GABA supplementation from many sources can improve the egg performance, immune response, and alleviate stress of laying hens. Zhang et al. [9] reported that supplementation of GABA as active ingredient form at 50 mg/kg diet improved the laying performance, egg production, and eggshell breaking strength in laying hens. Egg production, average egg weight, average daily feed intake, feed conversion ratio, percentage of speckled egg, soft shell egg, misshaped egg, and eggshell thickness were significantly improved by the increasing supplementation of a dietary GABA-producing Lactobacillus strain [10]. In addition, Jeong et al. [4] noted that dietary GABA as active ingredient form was effective in alleviating stress responses, but the effect was independent of stocking density in broilers. In our previous report, GPR contained 7.48% crude protein, 1.96% ether extract, 67.58% nitrogen free extract, and 12.55% crude fiber as dry matter basis. The gross energy of GPR was 3.77 kcal/g, which was found to be close to the corn. In addition, GPR exhibited a high content of phytonutrients, especially GABA, and this enhanced its antioxidant activities. Therefore, GPR could be a good alternative source of feed ingredients for poultry [11].

The emergence of novel proteomic techniques in recent years has greatly aided in the understanding of cell functions, physiological and biochemical pathways, and of biological mechanisms [12,13]. Proteomics can identify the gene that encodes the protein by combining the amino acid sequence with the genomic database and speculate on the function of the unknown protein. Biomarkers of stress in blood cells which are key components of the immune response, the coagulation cascade, and other functions such as iron and oxygen transportation and protection of blood vessels can be identified [14]. High-abundance proteins in serum include albumin, immunoglobulins, and transferrin [15]. Studies on stress proteins, encoded from white blood cells (WBC), have been related to many proteins such as inflammatory protein (NF-κB and TNF-α), interferon (IFN) [16] and heat shock protein (HSP) [17]. They also related to the reduction in antioxidant enzymes such as superoxide 4 dismutase (SOD-4), glutathione peroxidase (GPx), and malondialdehyde (MDA) [18]. It seems that WBC is a good indicator of stress. In addition, the highly complex protein mixtures can be analyzed for proteomic purposes by liquid chromatography coupled with tandem mass spectrometry (LC−MS/MS). Therefore, this study aimed to investigate the effect of dietary germinated paddy rice on egg performance, serum biochemical properties, and proteomic and transcriptomic response of laying hens reared in different stocking densities under chronic heat stress conditions.

## 2. Materials and Methods

### 2.1. GPR Preparation and GABA Content Analysis

Paddy rice variety (RD31) harvested during October 2018 was obtained from Phitsanulok province (Thailand). Paddy rice was germinated following the method described previously [11]. Briefly, 10 kg of paddy rice was soaked with water (1:2 *w*/*v*) at room temperature for 12 h, in which the water was changed every 4 h to avoid fermentation, and then drained. Soaked rice was spread in plastic basket with the maximum thickness of 2 cm and then covered with damp cloths maintaining the relative humanity of 90–95% by regular spraying of water. The germination took about 24 h. The GPR was dried at 50 °C in a tray-dryer, to approximately 10% of moisture content. The GABA content in GPR was determined using a high-performance liquid chromatography (HPLC; Shimadzu model LC-2010A, Shimadzu Corp., Kyoto, Japan) with a Kromasil 5 µm 100A C18 column (250 mm × 4.6 mm). The GABA contents was measured according to the method described previously with some modifications [19].

### 2.2. Birds and Experimental Design

Experimental procedures and animal care were carried out according to the animal regulation and guidelines of Naresuan University Agricultural Animal Care and Use Committee (NUAACUC; approval number: 6201002). Twenty-week-old Hy-line Brown laying hens were purchased from a commercial farm in Phitsanulok province, Thailand. They were maintained in wire cages in an environmentally controlled room with the stable photoperiod (17L:7D). A commercial laying diet (18% CP; 2800 kcal/kg) and drinking water were provided *ad libitum* during whole pre-experimental period. At forty two weeks, a total of 72 hens with the same egg uniformity were separated into 6 groups with four replicates per treatment. A completely randomized experimental design with 2 × 3 factorial arrangement was applied and consisted of the following factors: two stocking densities (LSD and HSD) and three dietary GPR levels (0, 74 and 148 g/kg), representing GABA content of 0, 3.1, and 6.3 mg/kg, respectively. Three groups of birds were reared in a wire cage (0.12 m^2^/bird) and expressed as LSD. The other three group were placed in a wire cage (0.06 m^2^/bird) and expressed as HSD. All experimental birds were kept in a same housing under daily cycling heat stress of 27–35 °C for 6 h, 35 ± 1 °C for 12 h, and 35–27 °C for 6 h. Relative humidity was kept approximately 65–70% during the entire experimental period. Each dietary treatment was randomly assigned to four replicate cages under each stocking density. All animals had free access to feed and water during 42 to 56 weeks of age. Dietary GPR levels were formulated to meet or exceed the NRC (1994) [20] and are displayed in Table 1.

### 2.3. Assessment of Egg Performance, Shell Hardness and GABA Content

All data were recorded during the whole 14-week experimental period to evaluate the egg performance and egg quality of laying hens reared in different stocking densities and fed different level of dietary GPR. Eggs were collected from each experimental cage 2 times per day (6:00 a.m. and 6:00 p.m.) and expressed as a number of eggs for one replication. Collected eggs were weighed and recorded daily, while remaining feed was measured weekly. Hen-day egg production, average egg weight, egg mass, average daily feed intake, feed conversion ratio were calculated on the basis of recorded data.

Shell breaking strength was evaluated using a TA-XT2 Plus Analyzer (Stable Microsystems, UK), following the methods described by Likittrakulwong et al. [21]. GABA content in yolk was measured using a modification of the method previously described [19].

### 2.4. Blood and Serum Samples Collection

Blood collections were performed on all hens (72 hens) at 0, 7, and 14 weeks; they were taken by venipuncture from the wing vein and blood saved into collection tubes using a sterile syringe and kept in blood collection tubes, then stored at 4 °C. Blood samples were mixed with anticoagulant solution [ethylene diamine tetraacetic acid; (EDTA)] and then used for gene expression analysis. For proteomic analysis and determination of biochemical parameters, hormone levels and immune activities, blood samples without anticoagulant solution were used. Blood samples were allowed to clot at 37 °C for 2 h to collect supernatant. Serum samples were centrifugated at room temperature for 15 min at 1000× *g* and then kept at −80 °C until used [22].

### 2.5. Serum Assay

The biochemical parameters and hormone levels comprised the levels of calcium (Ca), phosphorus (P), blood urea nitrogen (BUN), cholesterol (CHO), glucose (GLU), triglyceride (TG), albumin (ALB), corticosterone (CORT) hormone in serum samples, and were determined using an Olympus AU400 chemistry analyzer (Olympus Optical Co., Ltd., Tokyo, Japan) following the manufacturer’s instruction. Immune activities, including alternative complement haemolytic 50 (ACH50) activities, total immunoglobulin (Ig) and Lysozyme activities (LZY) were determined using a modification of the method previously described [23]. Antioxidant activities, SOD activities were measured in a direct dependency manner with reaction inhibition rate (%) of WST-1 substrate (a water-soluble tetrazolium dye) with xanthine oxidase using SOD Assay kit 19160 (Merck, Nottingham, UK).

### 2.6. Proteomic Analysis Using LC-MS/MS Technique

#### 2.6.1. Sample Preparation for Shotgun Proteomics

Protein concentration of 216 serum samples was determined by Lowry assay using BSA as a standard protein [24]. Samples were reduced disulfide bonds using 5 mM dithiothreitol (DTT) in 10 mM AMBIC at 60 °C for 1 h and alkylation of sulfhydryl groups by using 15 mM Iodoacetamide (IAA) in 10 mM AMBIC at room temperature for 45 min in the dark. For digestion, samples were mixed with 50 ng/µL of sequencing grade trypsin (1:20 ratio) (Promega, Germany) and incubated at 37 °C overnight. Prior to LC-MS/MS analysis, the digested samples must be dried and protonated with 0.1% formic acid before injection into LC-MS/MS.

#### 2.6.2. LC-MS/MS

The tryptic peptide samples were prepared for injection into an Ultimate3000 Nano/Capillary LC System (Thermo Scientific, Loughborough, UK) coupled to a hybrid quadrupole Q-Tof impact II™ (Bruker Daltonics, Billerica, MA, USA) equipped with a nano-captive spray ion source. Briefly, peptide digests (1 μL) were enriched on a µ-Precolumn 300 µm I.D. × 15 mm C18 Pepmap 100, 5 µm, 100 A (Thermo Scientific, UK), separated on a 75 μm I.D. × 15 cm and packed with Acclaim PepMap RSLC C18, 2 μm, 100 Å, nanoViper (Thermo Scientific, UK). The C18 column was enclosed in a thermostatted column oven set to 60 °C. Solvent A and B containing 0.1% formic acid in water and 0.1% formic acid in 80% acetonitrile, respectively were supplied on the analytical column. A gradient of 5–55% solvent B was used to elute the peptides at a constant flow rate of 0.30 μL/min for 30 min. Electrospray ionization was carried out at 1.6 kV using the CaptiveSpray. Nitrogen was used as a drying gas (flow rate about 50 L/h). Collision-induced-dissociation (CID) product ion mass spectra were obtained using nitrogen gas as the collision gas. Mass spectra (MS) and MS/MS spectra were obtained in the positive-ion mode at 2 Hz over the range (*m*/*z*) 150–2200. The collision energy was adjusted to 10 eV as a function of the *m*/*z* value. The LC-MS analysis of each sample was done in triplicate.

#### 2.6.3. Bioinformatics and Data Analysis

MaxQuant 1.6.6.0 was used to quantify the proteins in individual samples using the Andromeda search engine to correlate MS/MS spectra to the Uniprot *Gallus gallus* database [25]. Label-free quantitation with MaxQuant’s standard settings was performed: maximum of two miss cleavages, mass tolerance of 0.6 Dalton for main search, trypsin as digesting enzyme, carbamidomethylation of cystein as fixed modification, and the oxidation of methionine and acetylation of the protein N-terminus as variable modifications. Only peptides with a minimum of 7 amino acids, as well as at least one unique peptide, were required for protein identification. Only proteins with at least two peptides, and at least one unique peptide, were considered as being identified and used for further data analysis. Protein FDR was set at 1% and estimated by using the reversed search sequences. The maximal number of modifications per peptide was set to 5. As a search FASTA file, the proteins present in the *Gallus gallus* proteome downloaded from Uniprot. Potential contaminants present in the contaminants FASTA file that comes with MaxQuant were automatically added to the search space by the software. The MaxQuant ProteinGroups.txt file was loaded into Perseus version 1.6.6.0 [26], potential contaminants that did not correspond to any UPS1 protein were removed from the data set. Max intensities were log_2_ transformed and pairwise comparisons between conditions were done via t-tests. Missing values were also imputed in Perseus using constant value (zero). The visualization and statistical analyses were conducted using the MultiExperiment Viewer (MeV) in the TM4 suite software. Protein organization and biological action were investigated conforming to protein analysis through evolutionary relationships (Panther) protein classification [22]. Venn diagrams were used to show the differences between protein lists originating from different differential analyses [22]. The STITCH database version 5 was used to analyze the common and the forecasted functional interaction networks between identified proteins and small molecules [27]. The raw MS/MS spectra data are available in ProteomeXchange:JPST001371 and PXD029557; https://repository.jpostdb.org/preview/9132357196183ac3de0a81 (Access key 2730, accessed on 1 December 2021). (see Supplementary Materials Table S1).

### 2.7. Gene Expression Analysis

Total RNA was extracted from blood lymphocytes using a QIAamp RNA blood mini kit (Qiagen, Hilden, Germany). One microgram of total RNA from each group of laying hens collected at various time points was used for first-strand cDNA synthesis, which was performed using the RevertAid^TM^ first strand cDNA synthesis kit (Fermentas, Burlington, Canada), following the recommendation of the manufacturer. One microliter of first-strand cDNA from each sample was used as the template for semi-quantitative RT-PCR analysis. PCR amplification was performed using the specific primers (Table 2). Semi-quantitative RT-PCR was performed as previously described by Likittrakulwong et al. [28] to measure the levels of heat shock protein 70 and 90 (HSP70, HSP90), 3-hydroxyl-3-methyl-glutarl coenzyme A reductase (HMGCR), fatty acid synthase (FASN), fatty acid binding protein4 (FABP4) and beta-actin mRNAs. The reactions were performed in triplicate in a MyGo Pro real-time PCR instrument (IT-IS Life Science Ltd., Mahon, Cork, Ireland). The relative expression ratios of mRNAs of these genes were analyzed using MyGoPro qPCR software (IT-IS Life Science Ltd., Mahon, Cork, Ireland). The results of real-time PCR for gene transcripts were analyzed by the 2^−ΔΔct^ calculation [29]. The mRNA levels of these genes were corrected using the transcription level of the beta-actin gene as a housekeeping gene.

### 2.8. Statistical Analysis

Each cage represented an experimental unit. Data for all variables were analyzed by a two-way analysis of variance (ANOVA). The model including stocking density and dietary GPR levels as the main factors and their interaction was examined using the general linear model (GLM) procedure in SPSS version 23.0 (SPSS Inc., Chicago, IL, USA). For gene expression analysis, the relative expression ratio was determined with one-way ANOVA. Differences among the groups were analyzed using the Tukey test. Results were expressed in terms of mean ± SD.

## 3. Results

Previously published research has shown that the health and welfare of chickens are compromised if space allowances decrease 0.116 to 0.0578 m^2^/bird [5]. Thus, two levels of stocking densities (HSD and LSD; 0.06 and 0.12 m^2^/bird, respectively) were investigated in the current experiment.

### 3.1. Egg Performance, Shell Hardness and GABA Content

During the whole experimental period, hen-day egg production, egg mass, average daily feed intake, shell breaking strength, and GABA content in eggs were lower (*p* < 0.01) in HSD group than in the LSD group (Table 3). However, there were no significant differences in average egg weight between both treatments. Compared to the 0 g/kg GPR group, shell breaking strength was significantly increase (*p* < 0.01) in 74 and 148 g/kg GPR group, respectively. With increasing dietary GPR levels, GABA content in eggs tend to increase in dietary GPR groups and significantly increase (*p* < 0.01) in 148 g/kg GPR group. On the other hand, no significant differences in hen-day egg production, egg mass, average daily feed intake or average egg weight were observed between both treatments. There were, however, significant interactions (*p* < 0.05) between stocking density and dietary GPR levels for shell breaking strength (Table 3).

### 3.2. Biochemical Parameters and Hormone Contents

It was found that the levels of serum GLU, P, and CORT were increased (*p* < 0.05) in LSD versus HSD. The dietary GPR levels significantly affected (*p* < 0.01) GLU, P and CORT hormone levels. Compared with the other three groups, the significantly highest level of serum P was recorded in hens supplied with 148 g/kg GPR, 6.5 mg/dL (*p* < 0.05). In addition, the level of serum Ca tended to increase in chickens fed with dietary GPR levels, compared to the control (0 g/kg GPR) (Table 4). Conversely, the significantly lowest concentration of CORT in serum samples was (*p* < 0.01) found in hens fed with 148 g/kg GPR, 37.8 ng/dL. The levels of serum BUN, CHO, TG, ALB, and Ca were affected by stocking densities or dietary GPR levels (Table 4). The concentrations of GLU, P, and CORT hormone in serum samples showed that there were interactions (*p* < 0.05) between stocking densities and dietary GPR levels. The highest level of serum CORT was noted in T4, 357.5 ng/dL, (*p* < 0.01). Increasing dietary GPR levels significantly decreased corticosterone hormone.

### 3.3. Antioxidation and Immune Activity

In terms of stocking densities, the concentration of total Ig, LZY and SOD in serum samples were increased (*p* < 0.05) in LSD versus HSD (Table 5). The dietary GPR levels significantly affected (*p* < 0.01) ACH50, total Ig, LZY and SOD. The significantly highest levels of serum ACH50, total Ig and LZY and SOD were found in hens supplied with 74 g/kg GPR, at the level of 61.6 U/mL, 3.9 mg/mL, 1839.4 and 109.0 U/mL (*p* < 0.01), respectively. The ACH50, total Ig and SOD in serum samples showed that there was interaction (*p* < 0.01) between stocking densities and dietary GPR levels. The highest level of serum ACH50 and total Ig were found in T5 (66.3 U/mL and 4.0 mg/mL, respectively, while the highest SOD was observed in T6 (153.6 U/mL) (*p* < 0.01) (Table 5). Immune and antioxidant capacity were affected by stocking density or dietary GPR levels as well as the interactions between the two main factors.

### 3.4. LC-MS/MS Identification

**Figure 1:**
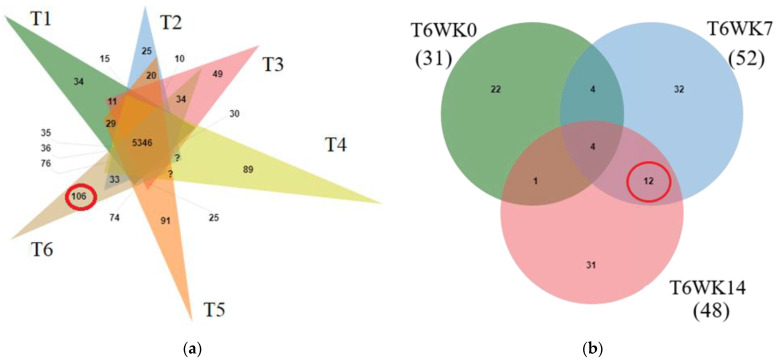
Overlap of identified and abundance protein. (**a**) Overlap of identified protein in all treatment T1 (green), T2 (pink), T3 (orange), T4 (blue), T5 (light green), and T6 (brown) (**b**) Overlap of abundance protein in Treatment 6 at different times (in WK0, T6WK0, green; in WK7, T6WK7, blue; in WK14, T6WK14, pink). GPR: germinated paddy rice; LSD: low stocking density; HSD: high stocking density; T1: LSD + 0 g/kg GPR; T2: LSD + 74 g/kg GPR; T3: LSD + 148 g/kg GPR; T4: HSD + 0 g/kg GPR; T5: HSD + 74 g/kg GPR; T6: HSD + 148 g/kg GPR; WK0: at 0 week (early interval times); WK7: at 7 week (middle interval times); WK14: at 14 week (late interval times).

To determine the protein composition changes in all treatments, peptide sequences from liquid chromatography-tandem mass spectrometry (LC-MS/MS) were investigated using the *Gallus gallus* genome database. From the total of 8607 proteins, detectable amounts of 6804, 6787, 6705, 7647, 7542, and 7685 predicted proteins were present in the T1, T2, T3, T4, T5, and T6 samples, respectively. A total of 5346 proteins were shared in all treatments. A total of 106 proteins were specifically presented in T6 (Figure 1a). Moreover, the total of 106 predicted proteins from T6 were evaluated for proteins changes during early, middle and late induction (0, 7 and 14 week; WK0, WK7, and WK14). It was found that amounts of 31, 52, and 48 proteins were present at all induction times (T6WK0, T6WK7, and T6WK14), respectively. Four proteins were shared in all stages, namely repressor of polymerase III transcription MAF1homolog (MAF1; A0A3Q2TX85), Calpain inhibitor (CAST; A1E134), TALPID3 protein (TALPID3; Q1G7G8), and transcription factor BTF3 (BTF3; F1NKT5). Twelve proteins were specifically shown in the middle and last (T6WK7 and T6WK14) but not in early induction times (T6WK0) (Figure 1b).

#### Differentially Abundant Protein in All Treatments during Induction Times

Abundant protein profile changes from all treatments during induction time (early, middle, and late; at 0, 7, and 14 week) were analyzed. Twelve proteins were specifically shown in middle and late (T6WK7 and T6WK14) but not in early induction times (T6WK0), namely conserved oligomeric Golgi complex subunit 1 (COG1; A0A1D5P6I3), homeodomain protein (NKx-6.2; Q9PTK0), tropomodulin (fragment) (E-Tmod; Q9DEA6), MHC class I antigen (MHCI; A0A089FGZ7), ABC transporter domain-containing protein (RCJMB04; Q5ZHK3), forkhead transcription factor L2 (FoxL2; Q7T269), Mdm4 protein (MDM4; A0A3Q2TVB9), neuronal PAS domain-containing protein 2 (NPAS2 MOP4;Q5ZQU2), CD3 epsilon chain (CD3e; B3VMQ8), gonadotrophin-releasing hormone-II (GnRH II; H6UQ73), lipopolysaccharide-binding protein (LOC419276; A0A1D5PT78), and apoptosis-associated protein (O42561) (Table 6). One protein was specifically presented in T4 at 14 week (T4WK14) but not in the others, namely telomerase reverse transcriptase isoform I (TERT; Q3YAE7). Low-density lipoproteins receptor-related proteins 2 (LRP2; G0W2S9) was specifically presented in T6 at week 14 (T6WK14). The highest abundance protein levels of HSP90, HSP70, HMGCR, FASN and FABP4 were in T2 at 0 week (T2WK0), T4 at 0 week (T4WK0), T5 at 7 week (T5WK7), T1 at 7 week (T1WK7), and T2 at 7 week (T2WK7), respectively (Table 6).

### 3.5. Quantitative Real-Time Reverse Transcription PCR (qPCR)

**Figure 2:**
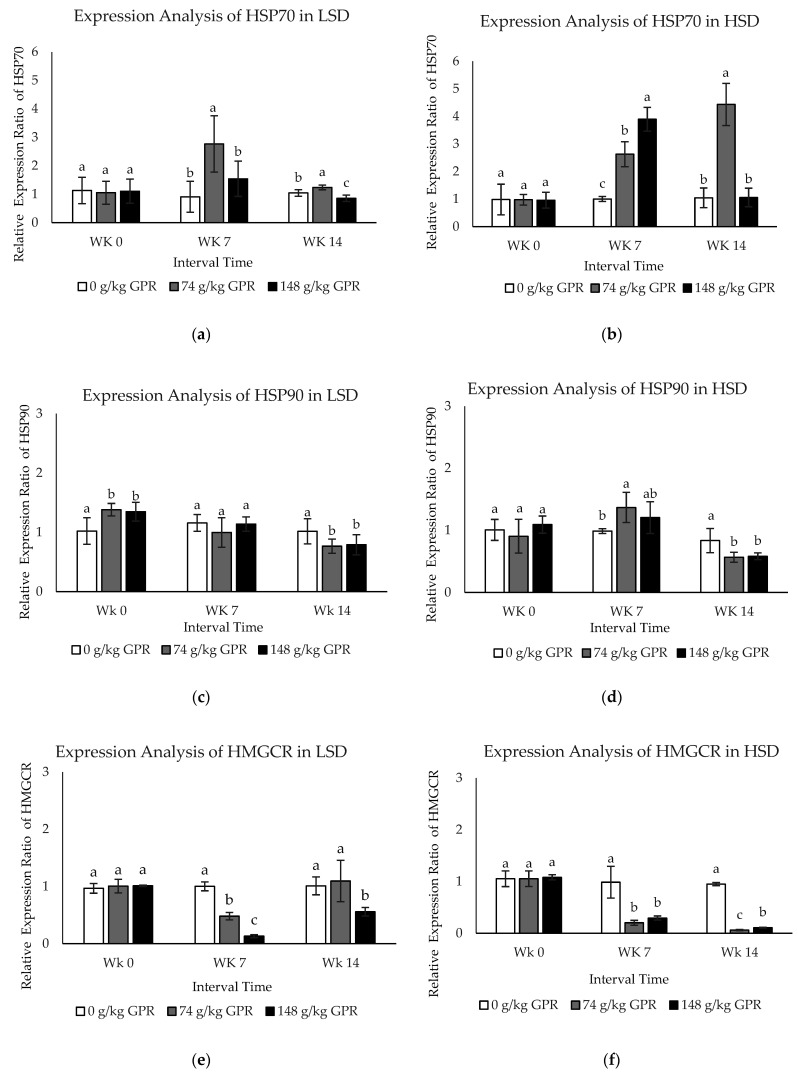

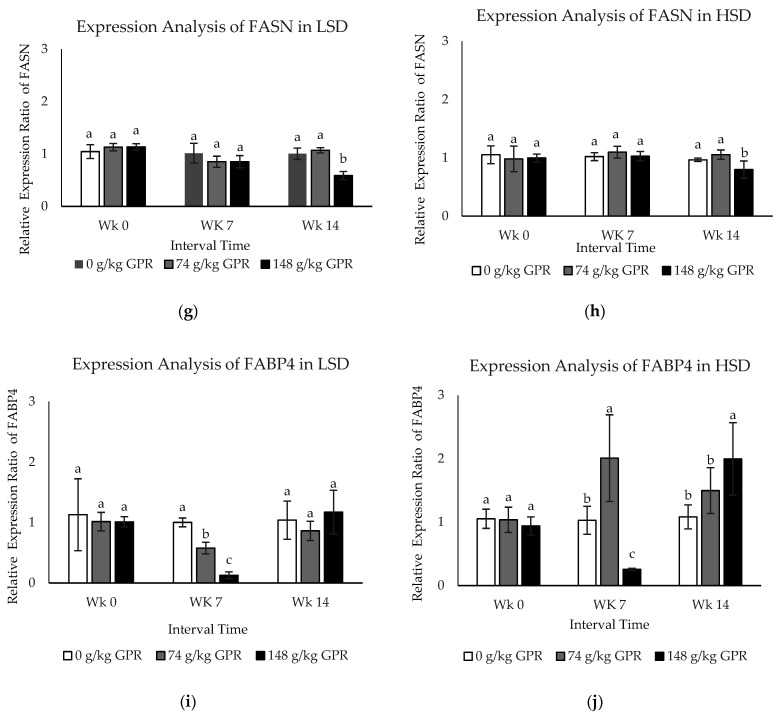
The mRNA expression levels in blood samples of layer hens after different stocking density and different GABA level from germinated paddy rice supplementation. (**a**,**c**,**e**,**g**,**i**) The mRNA expression levels of HSP70, HSP90, HMGCR, FASN, and FABP4 of laying hens that were fed experimental diets for 98 day in low stocking density (0.12 m^2^/bird). (**b**,**d**,**f**,**h**,**j**) The mRNA expression levels of HSP70, HSP90, HMGCR, FASN, and FABP4 of laying hens that were fed experimental diets for 98 days in high stocking density (0.06 m^2^/bird). Data are presented as mean ± SD. Different lowercase letters above each bar indicate significant different at the same time point (*p* < 0.05).White filled bar: Control (0 g/kg GPR); gray filled bar = 74 g/kg GPR; black filled bar = 148 g/kg GPR. Abbreviation: GPR = germinated paddy rice; HSP: heat shock protein; HMGCR: hydroxyl-3-methyl-glutaryl coenzyme A reductase; FASN: fatty acid synthase; FABP4: fatty acid binding protein 4; WK0: at 0 weeks (early interval times); WK7: at 7 weeks (middle interval times); WK14: at 14 weeks (late interval times.

To evaluate differentially expressed protein changes from all treatments during interval times, the qPCR technique was applied. In the present study, the mRNA expression levels of five differentially expressed genes (HSP70, HSP90, HMGCR, FASN, and FABP4) were investigated in response to different stocking densities and different dietary GPR levels during interval times (at 0, 7, and 14 weeks) (Figure 2). Stocking densities significantly affected (*p* < 0.05) the expression of HSP70, HSP90, HMGCR, FASN, and FASN. The expression levels of HSP70 and FABP4 in response to HSD were much higher than those with LSD.

The expression levels of HSP70 in each treatment in response to LSD at different times showed that expression of HSP70 was greatly induced during the middle interval times (WK7, *p* < 0.05). However, expression was clearly reduced during the late time interval (WK14, *p* < 0.05) in chickens fed the dietary at 74 g/kg GPR compared to the control (0 g/kg GPR) (Figure 2a). In addition, HSP70 expression levels in response to HSD were significantly up-regulated at WK7 but obviously down-regulated at WK14 for chickens fed with 148 g/kg GPR compared with the 0 g/kg GPR as shown in Figure 2b.

During the early interval time, HSP90 mRNA in response to LSD was slightly (*p* < 0.05) up-regulated in chickens supplied with 74 and 148 g/kg GPR, compared to the control group (0 g/kg GPR). However, expression was clearly reduced to the same levels as in the control group during the middle time (Figure 2c). HSP90 mRNA in response to HSD was up-regulated during middle interval times in chickens fed with 74 and 148 g/kg GPR, compared with the control (Figure 2d). Interestingly, during late interval times (WK14), both HSP90 mRNA in response to LSD and HSD were down-regulated in chickens fed with 74 and 148 g/kg GPR diets, compared with the control group. At WK7, HMGCR expression levels in response to LSD were slightly down-regulated, but moderately increased at the same levels of the control group at WK14 (*p* < 0.05) in chicken fed 74 g/kg GPR (Figure 2e). However, HMGCR expression levels in response to HSD were strongly decreased at 7 and 14 WK in chickens fed with 74 and 148 g/kg GPR compared with the control group (Figure 2f).

The expression levels of FASN mRNA in each treatment in response to LSD and HSD at the different times revealed that both FASN mRNA were significantly down-regulated at WK14 in chickens fed with 148 g/kg GPR, compared with the control (Figure 2g,h). During middle interval times, FABP4 expression levels in response to LSD were slightly down-regulated, but moderately increased to the same levels as in the control group during the last interval times in chickens fed with 74 and 148 g/kg GPR diets compared with the control diet (Figure 2i). Conversely, FABP4 expression levels in HSD were significantly induced during the middle time interval (WK7, *p* < 0.05) but clearly reduced during the late time interval (WK14, *p* < 0.05) at the same levels of the control group for chickens fed with 74 g/kg GPR compared with the control diet (Figure 2j). Increasing dietary GPR levels significantly induced expression levels of HSP70 and FABP4 but reduced expression levels of HSP90, HMGCR, and FASN.

## 4. Discussion

Studies on the physiological responses of poultry to stocking density suggested that certain physiological parameters, biochemical parameters, and expression genes such as GLU, CHO, TG, CORT, Ca, P), LYZ, antioxidant activities, HSP, HMGCR, FASN, and FABP4 could be used to evaluate the chickens under different stocking densities [4,5,9,10,32]. HSD affected growth performance, egg production, eggshell quality, health, and immunity [33]. HSD elevated CORT and heat shock protein 70 (HSP70) expression when compared to broilers raised at LSD [6].

In this study, increasing stocking densities reduced some parameters such as average daily feed intake, hen-day egg production, egg mass, shell breaking strength, and GABA content in eggs.

Several studies have illustrated the negative effect on body weight gain and feed intake of stocking densities. The review by Wang et al. [34] indicated that higher stocking densities tended to be associated with reduced feed intake and reduced weight gain in broilers. Jeong et al. [4] evaluated chickens raised on HSD, reporting that they exhibited a decrease in body weight gain in all phases, feed intake, and an increase in FCR compared with LSD-raised chickens. HSD might contribute to reduced broiler performance because of high temperatures and reduced airflow around the birds. Cage area might affect poultry production, which also affects locomotor activity [34]. In this study, it was observed that dietary GPR levels significantly improve shell breaking strength and GABA content in eggs. GPR consists of rice husk, which has a high ash content (14–25%). The silica content of the rice husk ash can be as high as 90–98%. Some evidence suggests the involvement of silicon in the formation and stabilization of the extracellular matrix as well as the Ca metabolism. In previous reports, rice hull silicon plays a major role in improving bone-breaking strength and reducing drip and thawing loss of broiler chicken breast and thigh muscles [35]. Moreover, whole rice hull can be used as a source of insoluble fiber in diets to enhance growth and uniformity of pullet chicks and to improve egg production of laying hens without any harmful impact on egg quality [36]. In addition, GPR contains phytonutrients, especially GABA, which enhance the antioxidant activities.

Similar findings have been documented by previous researchers. Chand et al. [37] reported that GABA supplement diets improved the feed intake, body weight, antioxidant status, and immune activity in broilers. El-Naggar et al. [38] investigated GABA supplementation-increased feed intake by reducing the mRNA expression levels of feed intake-inhibiting neuropeptides, such as proopiomelanocortin (POMC), leptin, chrelin, and cholescystokinin (CCK) by increasing the expression of feed intake-stimulating neuropeptides such as agouti-related protein (AgRP) and neuropeptide (NPY). Zhu et al. [10] reported that supplementation with 50 mg GABA/kg diet improved average daily feed intake, feed conversion ratio, average egg weight, percentage of speckled egg, soft shell egg, and misshapen egg by enhancing the calcium and phosphorus available for eggshell formation. Zhang et al. [9] reported that GABA diets in hens could increase amylase, lipase, and trypsin activities in the gastrointestinal tract, thereby increasing egg production, average egg weight, and average daily feed intake. Additionally, Chen et al. [39] reported that GABA could increase the activity of Ca^2+^-Mg^2+^-adenosine and Na^+^-K^+^-adenosine, benefiting the transportation of mineral element. Consequently, GABA improved egg production and egg quality by increasing the total protein concentration and modulating the electrolyte balance [9].

Serum biochemical parameters are the key indicators for the health of poultry. In this study, diet GPR levels trended to improve the levels of serum CHO, TG and Ca and significantly improved the concentration of GLU, P and CORT hormone (Table 4). Similar findings have been reported elsewhere. For example, Houshmand et al. [40] identified increases in GLU and CHO in HSD-raised chickens. Zhigang et al. [41] reported that levels of serum CHO and TG were increased in cherry valley duck supplemented with 100 mg GABA/kg diet. Curiously, GABA supplementation in chickens reduced lipid deposition and decreased abdominal fat contents, despite the growth in serum triglyceride. This effect may have been due to increased serum levels of GABA, which can stimulate the turnover of fat and the release of free fat acids and glucose into serum to be available to all cells as energy sources [32].

In contrast, Jeong et al. [4] noted that blood glucose was decreased in HSD-raised chickens. In addition, CORT is a commonly stress hormone which measured in chickens. In general, the current results confirmed that finding. The levels of serum CORT were higher in chickens raised at HSD than those raised at LSD [6]. Increasing dietary GPR levels significantly decreased corticosterone hormone. Freeman and Crapo [42] reported that stress could stimulate the release of CORT, leading to overproduction of oxygen free radicals OH and O_2_. However, the body is protected by antioxidant defense systems working in associated with intracellular enzymes such as SOD. SOD in the mitochondria of cells functions to remove superoxide anions by transforming them into H_2_O_2_ and O_2_ [43]. It should be noted that the levels of total Ig, LZY, and SOD activities were statistically higher in chickens raised at HSD than those in LSD. Dietary at 74 g/kg GPR improved the level of serum ACH50, total Ig, LZY, and SOD activities (Table 5). Our results are in agreement with Zhang et al. [9] who reported that GABA significantly improved immune and antioxidant activity by increasing ACH50, total Ig, LZY, and SOD activities. The synthesis of antibodies was improved by adding 50 mg GABA/kg feed, which protected immune cells from the over-production of reactive oxygen species (ROS) caused by heat stress due to the activation of the antioxidant defense system [10].

An abundance of differential protein in all treatments at different times was demonstrated (Table 6). Twelve proteins were present only in T6 during WK7 and WK14 (chickens raised in HSD and dietary at 148 GPR at the middle and late induction times). One protein (TERT; Q3YAE7) was present only in T4WK14 (chickens raised in HSD and dietary 0 g/kg GPR at 14 weeks). One protein (LRP2; G0W2S9) was present only in T6WK14 (chickens raised in HSD and dietary at 148 g/kg GPR at 14 weeks). In addition, we identified four characterized proteins including HSP90, HSP70, HMGCR, FASN, and FABP4 (Table 6). Telomerase is a ribonucleoprotein enzyme that adds telomeric repeats on telomeres, which are nucleoprotein structures at the ends of eukaryotic chromosomes consisting of tandem repeats of TTAGGG sequences. Telomeres are highly sensitive to damage induced by oxidative stress [44]. Therefore, telomeric DNA length and telomerase activity are good candidate genes for physiological stress indicators. Sohn et al. [45] reported that telomere-shortening rate increased in chickens raised under HSD as compared with LSD. HSD had a negative effect on shortening telomere length [5]. The heat shock proteins (HSP) are a group of conserved proteins that are expressed under thermal and non-thermal stressors. HSP might be stimulated via physiological stresses, pathological stresses, and environmental stresses (such as heat or cold stress, oxidative stress, stocking density stress). HSPs are involved in protein synthesis, and can be classified into several subgroups of molecular chaperones according to their molecular weights (small HSPs, HSP60s, HSP70, HSP90s, and HSP100s). HSPs are considered the protective agents against stress factors [46]. HMGCR is the rate-controlling enzyme in cholesterol biosynthesis, which in turn is a precursor of cortisol, a universal stress marker. Therefore, increasing cortisol levels in high stocking density can cause negative impact with the synthesis of HMGCR gene under stress situations [5]. FASN is a key enzyme in fatty acid synthesis which catalyzes the synthesis of long-chain fatty acids. The conversion of acetyl-CoA into malonyl-CoA and then into palmitate is mediated by FASN and ends in triglyceride formation [47] FABP4 also plays an important role in lipid accumulation during adipogenesis [48]. It is considered to be important during adipose development because of its role in sequestering fatty acids for triacylglycerol synthesis and as a signaling molecule that regulates activity of enzymes in the adipocyte. Curiously, dietary GPR level supplementation dramatically affected the expression levels of the fat metabolism-related genes *FAS* and FABP4, and this impact was linked to reduced fat accumulation in the abdomen of the laying hens. This effect can stimulate the turnover of fat and release of free fat acid and glucose in serum to be available to all cells as energy sources.

To confirm the mRNA transcription level of identified proteins, the candidate genes were verified by using qPCR. The transcription levels of five genes (HSP70, HSP90, HMGCR, FASN, and FABP4) were determined (Figure 2). Beloor et al. [5] reported that the expression levels of HSP70 and HMGCR in blood were increased in HSD (0.0578 m^2^/birds) but were not significantly different compared with the LSD (0.116 m^2^/birds) and standard (0.077 m^2^/birds) groups. However, the stocking density did not affect HSP90. GABA significantly altered FASN gene expression, resulting in significant increase in abdominal fat content in chickens raised normally [38]. However, the mRNA and protein abundances did not always correlate well. It was often noted that the expression was conducted at the mRNA level, but not at the protein level, because of different regulation operating at transcription and translational steps [49,50].

**Figure 3:**
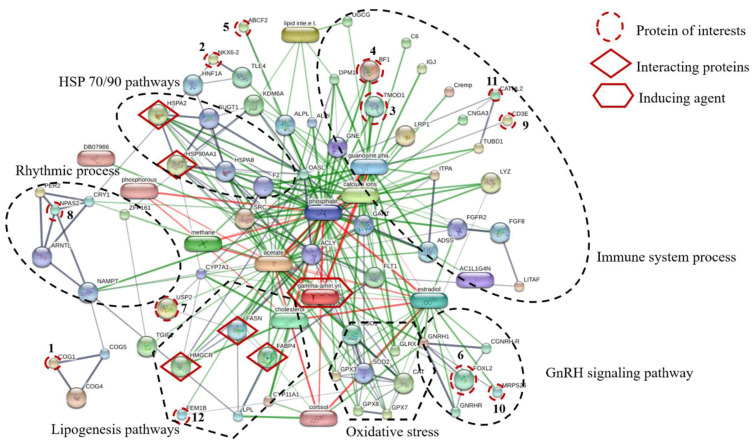
The network of identified twelve protein of interests and five interacting proteins predicted by STITCH database based on the following analysis parameters; species (*Gallus gallus*); medium confidence score (0.4) and active prediction methods (no more than 10 interactions). Twelve protein of interests, namely (1) conserved oligomeric Golgi complex subunit 1(COG1), (2) homeobox protein Nkx-6.2 (NKX6.2), (3) tropomodulin-1 (TMOD1), (4) MHC BF2 class I precursor (BF1), (5) ATP-binding cassette sub-family F member 2 (ABCF2), (6) forkhead box protein L2 (FOXL2), (7) ubiquitin carboxyl-terminal hydrolase 2 (USP2), (8) neuronal PAS domain-containing protein 2 (NPAS2), (9) T-cell surface glycoprotein CD3 epsilon chain precursor (CD3E), (10) mitochondrial ribosomal protein S26 (MRPS26), (11) cathelicidin-2 (CATHL2) and (12) stimulates the secretion of gonadotropins Protein fem-1 homolog B (FEM1B); five interacting proteins, including (1) heat shock 70 kDa protein (HSPA2), (2) heat shock protein 90 kDa alpha (cytosolic) class A member 1 (HSP90AA1), (3) 3-hydroxy-3-methylglutaryl-Coenzyme A reductase (HMGCR), (4) fatty acid synthase (FASN) and (5) fatty acid-binding protein (FABP4) and inducing agent (Gamma aminobutyric acid; gamma-amin.yri.). Abbreviation: Low-density lipoprotein receptor-related protein 1 precursor (LRP1), lipoprotein lipase (LPL), conserved oligomeric Golgi complex subunit 4, conserved oligomeric golgi complex subunit 5 (COG5), heat shock cognate 71 kDa protein (HSPA8), homeobox protein AKR (TGIF1), catalase (CAT), superoxide dismutase1 (SOD1), glutathione peroxidase 3 (GPX3), glutathione peroxidase 7 (GPX7), glutathione peroxidase 8 (GPX8), glutaredoxin-1 (GLRX), immunoglobulin J polypeptide, linker protein for immunoglobulin alpha and mu polypeptides precursor (IGJ), complement component C6 precursor (C6), complement component 4 binding protein, alpha chain precursor (Cremp), zinc finger protein 161 homolog (ZFP161), proto-oncogen tyrosine-protein kinase Src (DB07966), adenylosuccinate synthetase isozyme 2 (ADSS), lipid intermediate II, alkaline phosphatase, tissue-nonspecific isozyme precursor (ALPL), alkaline phosphatase (ALPI), zinc finger protein 161 homolog (ZFP161), hepatocyte nuclear factor 1-alpha (HNF1A), suppressor of G2 allele of SKP1 homolog (SUGT1), prothrombin precursor (F2), transducin-like enhancer protein 4 (TLE4), progonadoliberin-1 gonadoliberin-1 GnRH-associated peptide 1(GNRH1), gonadotropin-releasing hormone receptor (GNRHR), cholesterol side-chain cleavage enzyme (CYP11A1), cryptochrome-1(CRY1), period circadian protein homolog 2 (PER2), nicotinamide phosphoribosyltransferase (NAMPT), vascular endothelial growth factor receptor 1 (FLT1), fibroblast growth factor 8 precursor (FGF8), 59 kDa 2′-5′-oligoadenylate synthase-like protein (OASL), vyclic nucleotide-gated channel cone photoreceptor subunit alpha (CNGA3), inosine triphosphate pyrophosphatase (ITPA), uncharacterized protein (TUBD1), uncharacterized protein (UGCG), uncharacterized protein (DPM1), uncharacterized protein (KDM6A), calcium ions, phosphorous, phosphate, cholesterol, estradiol, guanosine diphosphate, acetate.

Proteins, protein interactions, and small molecules induced by GABA play major roles in the understanding of molecular and cellular functions. The association between 12 proteins of interest and five interacting proteins (HSP90, HSP70, HMGCR, FASN, and FABP4) were predicted by STITCH version 5.0 using the parameters followed as the organism of *Gallus gallus*, medium confidence score (0.4) and active prediction methods (no more than 10 interactions). The results showed that these proteins are associated with proteins involved in HSP70/90 pathways, immune system processes, GnRH signaling pathway, oxidative stress, lipogenesis pathways, and rhythmic process (Figure 3). The conserved oligomeric Golgi complex subunit 1 (COG1) was associated with COG4, COG5, nicotinamide phosphoribosyltransferase (NAMPT) and neuronal PAS domain-containing protein 2 (NPAS2) which were important in the rhythmic process. The homeobox protein Nkx-6.2 (NKX6.2) was linked to transducin-like enhancer protein 4 (TLE4), hepatocyte nuclear factor 1-alpha (HNF1A) and heat shock 70 kDa protein (HSPA2). Moreover, ATP-binding cassette sub-family F member 2 (ABCF2) was linked to tissue-nonspecific isozyme precursor (ALPL), uncharacterized protein (KDM6A), 59 kDa 2′-5′-oligoadenylate synthase-like protein (OASL), suppressor of G2 allele of SKP1 homolog (SUGT1), heat shock 70 kDa protein (HSPA2), heat shock cognate 71 kDa protein (HSPA8), heat shock protein 90 kDa alpha (cytosolic) class A member 1 (HSP90AA1) and also associated with transducin-like enhancer protein 4 (TLE4) which is known to be involved in HSP70/90 pathways. The forkhead box protein L2 (FOXL2) interacted with mitochondrial ribosomal protein S26 (MRPS26) which is known to be involved in GnRH signaling pathway. Tropomodulin-1 (TMOD1) interacted with MHC BF2 class I precursor (BF1), guanosine diphosphate, low-density lipoprotein receptor-related protein 1 precursor (LRP1), cathelicidin-2 (CATHL2) and T-cell surface glycoprotein CD3 epsilon chain precursor (CD3E), Guanosine phosphate, Calcium ions and GABA which are relevant in the immune system process. Ubiquitin carboxyl-terminal hydrolase 2 (USP2) was associated with FASN and stimulates the secretion of gonadotropins protein fem-1 homolog B (FEM1B), HMGCR, cholesterol, FABP4, lipoprotein lipase (LPL), cortisol hormone and GABA. The mechanism is related to the lipogenesis pathway. In addition, FEM1B was also associated and interacted with catalase (CAT), superoxide dismutase1 (SOD1) and GABA, which are known to be involved in oxidative stress. In this study, dietary GPR levels might affect growth metabolism, lipogenesis, oxidative stress, and immune defense mechanism of laying hens raised at different stoking densities.

## 5. Conclusions

We identified the proteins and the mRNA expression levels in all treatments at different times. The proteins identified in this study serve as a good candidate as biomarkers for chickens fed with dietary GPR levels under various stocking density conditions. There were 12 interacting proteins of interest, five interacting proteins and GABA molecule predicted by STITCH program. It indicated that stocking densities and dietary GPR levels could affect growth metabolism, lipogenesis, oxidative stress, and immune defense mechanism in laying hens.

Furthermore, dietary GPR could improve shell breaking strength, GABA content in eggs, CORT, immune and antioxidant activity, proteins, and gene expression levels without a negative effect on egg performance. In conclusion, the 74 g/kg dietary GPR were optimum for laying hens raised with high stocking density under heat stress conditions.

## Figures and Tables

**Table 1:** Ingredients and nutrient composition of the basal diets (as-fed basis).

Item	Dietary GPR, g/kg		
	0	74	148
Ingredients			
Corn	460.0	460.0	460.0
Paddy rice	148.0	74.0	-
GPR	-	74.0	148.0
Palm oil	44.0	44.0	44.0
Soybean meal, 460 g/kg CP	170.0	170.0	170.0
Fish meal, 600 g/kg CP	60.0	60.0	60.0
Calcium carbonate	99.0	99.0	99.0
Dicalcium phosphate, 180 g/kg P	11.0	11.0	11.0
Vitamin-mineral premix ^1^	3.0	3.0	3.0
DL-Methionine	2.0	2.0	2.0
Pigments	2.0	2.0	2.0
Salt	1.0	1.0	1.0
Total	1000	1000	1000
Calculated chemical composition ^2^			
Metabolizable energy, kcal/kg	2750	2750	2750
Crude Protein	165.0	165.0	165.0
Ether extract	69.5	69.5	69.5
Crude Fiber	40.2	40.2	40.2
Calcium	45.0	45.0	45.0
Available phosphorus	3.9	3.9	3.9
GABA content, mg/kg	0.0	3.1	6.3

GPR: germinated paddy rice, GABA: γ-amino butyric acid. ^1^ Vitamin-mineral premix provided per kilogram of diet: vitamin A, 10,000 IU; vitamin D3, 25,000 IU; vitamin E, 3.36 mg; vitamin K3, 2.00 mg; vitamin B1, 1.00 mg; vitamin B2, 3.00 mg; vitamin B6, 3.00 mg; vitamin B12, 0.02 mg; nicotinic acid, 20 mg; pantothenic acid, 5.00 mg; folic acid, 1.00 mg; biotin, 0.10 mg; choline chloride, 1.00 mg; selenium, 2.00 mg; cobalt, 1.00 mg; iodine, 1.00 mg; zinc, 50.00 mg; iron, 50.00 mg; manganese, 78.00 mg; copper, 4.00 mg. ^2^ The nutrient values were calculated based on the analyzed nutrient values according to NRC (1994).

**Table 2:** Specific primers used in the experiments.

Gene ^1^	Sequence (5′-3′)	Annealing Temperature (°C)	Product Size (bp)	Ref.
HPS70	F:AATCTATCATCATGTCTGGCAAAGGGCCGG	58 °C	220 bp	[5]
	R:GCGGCCGATGAGACGCTTGGCATCAAAGAT			
HPS90	F:ATGCCGGAAGCTGTGCAAACACAGGACCAA	55 °C	242 bp	[5]
	R:GGAATCAGGTTAATTTTCAGGTCTTTTCCA			
HMGCR	F:ATGCATGGCCTTTTTGTGGCCTCTCATCCA	55 °C		[5]
	R:CTTGAGAAGATTGTGAGGAGACCAGCAATA			
FASN	F:TTCGTGTTACCGCCTCAG	55 °C	91 bp	[30]
	R:TTCCCACTGCCTGCTTAG			
FABP4	F:ATGGCAAAGAGACTGTTATCAA	55 °C	118 bp	[30]
	R-TGAAGACGGCTTCCTCAT			
Beta-actin	F-CCACCGCAAATGCTTCTA	60 °C	96 bp	[31]
	R-GCCAATCTCGTCTTGTTTTATG			

^1^ HSP: heat shock protein; HMGCR: hydroxyl-3-methyl-glutaryl coenzyme A reductase; FASN: fatty acid synthase and FABP4:fatty acid binding protein 4.

**Table 3:** Effect of dietary GPR levels on egg performance and egg quality in laying hens during 42 to 56 week of ages.

Item			ADFI, g/b	AEW, g	HD, %	EM, g/b/d	SS, gf/m^2^	GABA in Egg, mg/100 g
Treatment	Stocking density	GPR, g/kg						
T1	LSD	0	96.9	46.7	80.5	37.6	2657.4 ^b^	9.5
T2		74	93.3	51.7	82.2	42.5	2732.6 ^b^	10.3
T3		148	91.5	50.3	82.0	41.2	3454.6 ^a^	12.8
T4	HSD	0	83.7	47.0	76.6	36.0	2062.0 ^c^	8.4
T5		74	82.6	48.0	76.0	36.5	2553.6 ^b^	8.9
T6		148	82.0	49.5	73.6	36.4	2795.1 ^b^	12.1
Pooled SEM			1.32	0.67	0.86	0.73	91.74	0.37
Main effect								
Stocking density								
LSD			93.9 ^a^	49.5	81.5 ^a^	40.4 ^a^	2948.2 ^a^	10.9 ^a^
HSD			82.8 ^b^	48.1	75.4 ^b^	36.2 ^b^	2470.2 ^b^	9.8 ^b^
GPR, g/kg								
0			90.3	46.8	78.5	36.8	2360.0 ^c^	8.9 ^b^
74			87.9	49.8	79.1	39.5	2643.1 ^b^	9.6 ^b^
148			86.7	49.9	77.8	38.8	3124.9 ^a^	12.4 ^a^
Effect (*p*-value)								
Stocking density			<0.010	0.287	<0.010	<0.010	<0.010	<0.010
GPR			0.067	0.103	0.682	0.157	<0.010	<0.010
Stocking density × GPR			0.447	0.417	0.324	0.272	0.036	0.668

GPR: germinated paddy rice; LSD: low stocking density; HSD: high stocking density; T1: LSD + 0 g/kg GPR; T2: LSD + 74 g/kg GPR; T3: LSD + 148 g/kg GPR; T4: HSD + 0 g/kg GPR; T5: HSD + 74 g/kg GPR; T6: HSD + 148 g/kg GPR; ADFI: average daily feed intake; AEW: average egg weight; HD: hen-day egg production; EM: egg mass; SS: shell breaking strength; SEM: standard error of the means. ^a–c^ Means with different superscripts in a column differ significantly (*p* < 0.05).

**Table 4:** Effect of dietary GPR levels on biochemical parameter of serum in laying hens during 42 to 56 week of ages.

Item			BUN, mg/dL	GLU, mg/dL	CHO, mg/dL	TG, mg/dL	ALB, g/dL	Ca, mg/dL	P, mg/dL	CORT, ng/dL
Treatment	Stocking density	GPR, g/kg								
T1	LSD	0	2.8	179.5 ^c^	126.8	763.3	1.9	18.0	3.3 ^b^	212.5 ^b^
T2		74	2.5	185.5 ^c^	138.0	997.0	2.0	22.4	7.1 ^a^	38.0 ^cd^
T3		148	3.0	210.3 ^b^	153.3	1001.0	2.0	20.9	6.2 ^a^	8.1 ^d^
T4	HSD	0	2.3	246.3 ^a^	111.8	682.5	2.0	16.1	6.7 ^a^	357.5 ^a^
T5		74	2.5	194.8 ^bc^	138.5	796.3	2.0	18.5	6.8 ^a^	65.0 ^c^
T6		148	2.5	197.0 ^bc^	116.8	858.0	2.0	18.4	6.8 ^a^	67.5 ^c^
Pooled SEM			0.10	5.28	9.90	62.40	0.04	1.09	0.36	26.27
Main effect										
Stocking density										
LSD			2.8	191.8 ^b^	139.3	920.4	2.0	20.4	5.5 ^b^	86.2 ^b^
HSD			2.4	212.7 ^a^	122.3	778.9	2.0	17.6	6.8 ^a^	163.3 ^a^
GPR, g/kg										
0			2.5	212.9 ^a^	119.3	722.9	1.9	17.0	5.0 ^b^	285.0 ^a^
74			2.5	190.1 ^b^	138.3	896.6	2.0	20.5	7.0 ^a^	51.5 ^b^
148			2.8	203.6 ^ab^	135.0	929.5	2.0	19.6	6.5 ^a^	37.8 ^b^
Effect (*p*-value)										
Stocking density			0.120	<0.010	0.437	0.290	0.837	0.234	0.032	<0.010
GPR			0.526	0.020	0.744	0.396	0.661	0.449	0.020	<0.010
Stocking density × GPR			0.526	<0.010	0.780	0.931	0.765	0.937	0.027	<0.010

GPR: germinated paddy rice; LSD: low stocking density; HSD: high stocking density; T1: LSD + 0 g/kg GPR; T2: LSD + 74 g/kg GPR; T3: LSD + 148 g/kg GPR; T4: HSD + 0 g/kg GPR; T5: HSD + 74 g/kg GPR; T6: HSD + 148 g/kg GPR; BUN: blood urea nitrogen; GLU: glucose, CHO: cholesterol; TG: triglyceride; ALB: albumin; Ca: calcium; P: phosphorus; CORT: corticosterone; SEM: Standard error of the means. ^a–d^ Means with different superscripts in a column differ significantly (*p* < 0.05).

**Table 5:** Effect of dietary GPR levels on serum parameters of immune and antioxidant activity in laying hens during 42 to 56 week of ages.

Item			ACH50, U/mL	Total Ig, mg/mL	LZY, U/mL	SOD, U/mL
Treatment	Stocking density	GPR, g/kg				
T1	LSD	0	59.4 ^ab^	3.0 ^d^	1014.4	105.4 ^b^
T2		74	56.8 ^ab^	3.8 ^b^	1733.3	99.0 ^b^
T3		148	37.4 ^c^	3.4 ^c^	1257.5	110.4 ^b^
T4	HSD	0	36.0 ^c^	4.1 ^a^	1056.3	105.0 ^b^
T5		74	66.3 ^a^	4.0 ^a^	1945.4	119.0 ^b^
T6		148	53.3 ^b^	4.0 ^a^	1266.7	153.6 ^a^
Pooled SEM			2.66	0.08	74.07	4.58
Main effect						
Stocking density						
LSD			51.2	3.4 ^b^	1335.1 ^b^	104.9 ^b^
HSD			51.9	4.0 ^a^	1422.8 ^a^	125.9 ^a^
GPR, g/kg						
0			47.7 ^b^	3.6 ^c^	1035.3 ^c^	105.2 ^b^
74			61.6 ^a^	3.9 ^a^	1839.4 ^a^	109.0 ^b^
148			45.3 ^b^	3.7 ^b^	1262.1 ^b^	132.0 ^a^
Effect (*p*-value)						
Stocking density			0.816	<0.010	0.048	<0.010
GPR			<0.010	<0.010	<0.010	<0.010
Stocking density × GPR			<0.010	<0.010	0.128	0.024

GPR: germinated paddy rice; LSD: low stocking density; HSD: high stocking density; T1: LSD + 0 g/kg GPR; T2: LSD + 74 g/kg GPR; T3: LSD + 148 g/kg GPR; T4: HSD + 0 g/kg GPR; T5: HSD + 74 g/kg GPR; T6: HSD + 148 g/kg GPR; ACH50: Alternative complement haemolytic 50; Total Ig: Total immunoglobulin; LZY: Lysozyme activities; SOD: Superoxide dismutase activities; SEM: Standard error of the means. ^a–d^ Means with different superscripts in a column differ significantly (*p* < 0.05).

**Table 6:** The list of differentially abundance protein in all treatments during different times.

	Uniprot Accession Number	Protein Name	Unique Peptide Sequences	Number of Unique Peptide	Unique Sequence Coverage (%)	Q-Value	Log_2_ Abundance of Protein ^1^																	
							WK0			WK7			WK14			WK0			WK7			WK14		
							T1	T2	T3	T1	T2	T3	T1	T2	T3	T4	T5	T6	T4	T5	T6	T4	T5	T6
1.	A0A1D5P6I3	Conserved oligomeric Golgi complex subunit 1	LDADCERVETR	1	1.2	1.00	0	0	0	0	0	0	0	0	0	0	0	0	0	0	11.72	0	0	13.42
2.	Q9PTK0	Homeodomain protein	AEPGALK	10	26.6	1.00	0	0	0	0	0	0	0	0	0	0	0	0	0	0	11.93	0	0	14.90
3.	Q9DEA6	Tropomodulin	ACAEALKTNTYVK	1	4.1	1.00	0	0	0	0	0	0	0	0	0	0	0	0	0	0	12.91	0	0	12.82
4.	A0A089FGZ7	MHC class I antigen	GITGDELIDCGSMWQVTHSEGTQNRRR	1	7.8	1.00	0	0	0	0	0	0	0	0	0	0	0	0	0	0	13.11	0	0	15.70
5.	Q5ZHK3	ABC transporter domain-containing protein	EAERLAHEDAECEKLMEFYER	4	14.1	1.00	0	0	0	0	0	0	0	0	0	0	0	0	0	0	13.34	0	0	13.12
6.	Q7T269	Forkhead transcription factor L2	KPPYSYVALIAMAIR	4	28.2	1.00	0	0	0	0	0	0	0	0	0	0	0	0	0	0	13.51	0	0	11.75
7.	A0A3Q2TVB9	Protein Mdm4	MTSSSSAQHPAAENACR	2	7.2	1.00	0	0	0	0	0	0	0	0	0	0	0	0	0	0	13.94	0	0	15.82
8.	Q5ZQU2	Neuronal PAS domain-containing protein 2	DSGSSLDPEQHFNALDIGASILSASR	2	6.4	1.00	0	0	0	0	0	0	0	0	0	0	0	0	0	0	14.24	0	0	17.16
9.	B3VMQ8	CD3 epsilon chain	AAAGSRPRAQKMQRPPPVPNPDYEPIR	2	16.6	0.99	0	0	0	0	0	0	0	0	0	0	0	0	0	0	14.42	0	0	12.12
10.	H6UQ73	Gonadotrophin releasing hormone-II	DQAEKRSQVVER	1	2.2	0.99	0	0	0	0	0	0	0	0	0	0	0	0	0	0	15.04	0	0	15.39
11.	A0A1D5PT78	Lipopolysaccharide-binding protein	DWSLPYHSGSSR	3	6.6	1.00	0	0	0	0	0	0	0	0	0	0	0	0	0	0	15.48	0	0	15.67
12.	O42561	Apoptosis associated protein	FGELTGGVTNAFCR	4	73.2	1.00	0	0	0	0	0	0	0	0	0	0	0	0	0	0	16.60	0	0	16.29
13.	Q3YAE7	Telomerase reverse transcriptase isoform I	LILRVHGIELINNHLMQLFFTFLT	2	60	0.99	0	0	0	0	0	0	0	0	0	0	0	0	0	0	0	13.05	0	0
14.	G0W2S9	Low-density lipoprotein receptor-related protein-2	HCNISHCAALSCQYRC	1	5	1.00	0	0	0	0	0	0	0	0	0	0	0	0	0	0	0	0	0	14.37
15.	A0A1D5P5R0	Heat shock protein HSP 90-alpha	DKEEVFRLIPYGIFQSK	1	5.3	1.00	0	12.73	0	0	0	0	0	0	9.49	0	0	0	0	0	12.12	11.89	10.97	0
16.	O73885	Heat shock cognate 71 kDa protein (Heat shock 70 kDa protein 8)	ARFEKLNADLFR	11	21.5	0.99	15.05	13.23	0	14.10	15.67	0	0	0	0	16.76	15.70	0	15.54	11.50	12.01	0	0	13.62
17.	V9IPH9	3-hydroxy-3-methylglutaryl-coenzyme A reductase	LGVQGASQDNPGENAR	2	85	0.99	0	0	0	0	0	0	0	0	0	12.65	0	12.36	15.34	15.39	0	0	0	12.36
18.	P12276	Fatty acid synthase	AGVAFHSYYMASIAPALLSALK	9	4.8	0.99	0	0	0	18.282	14.97	13.93	0	0	0	0	0	0	0	0	0	11.38	0	0
19.	A0A0N7G7I8	Adipocyte-type fatty acid-binding protein	CDQFVGTWK	2	27.3	0.99	0	0	12.41	0	15.98	0	11.94	0	0	13.77	0	0	0	14.20	12.68	14.67	0	12.74

^1^ The level of proteins in each samples were presented as log_2_ value; GPR: germinated paddy rice; LSD: low stocking density; HSD: high stocking density; T1: LSD + 0 g/kg GPR; T2: LSD + 74 g/kg GPR; T3: LSD + 148 g/kg GPR; T4: HSD + 0 g/kg GPR; T5: HSD + 74 g/kg GPR; T6: HSD + 148 g/kg GPR; WK0: at 0 week (early interval times); WK7: at 7 week (middle interval times); WK14: during at 14 week (late interval times).

## Data Availability

The datasets used and/or analyzed during the current study are available from the corresponding author on reasonable request.

## Supplementary Materials

The followings are available online at https://www.mdpi.com/article/10.3390/proteomes9040048/s1, Table S1: Information about the raw MS/MS spectra data.
